# Hedgehog/GLI Signaling Pathway: Transduction, Regulation, and Implications for Disease

**DOI:** 10.3390/cancers13143410

**Published:** 2021-07-07

**Authors:** Ashley N. Sigafoos, Brooke D. Paradise, Martin E. Fernandez-Zapico

**Affiliations:** Schulze Center for Novel Therapeutics, Division of Oncology Research, Mayo Clinic, Rochester, MN 55905, USA; Sigafoos.Ashley@mayo.edu (A.N.S.); Paradise.Brooke@mayo.edu (B.D.P.)

**Keywords:** hedgehog, cancer, GLI, SUFU, canonical and non-canonical activation, Hh pathway inhibitors

## Abstract

**Simple Summary:**

The Hedgehog/GLI (Hh/GLI) pathway plays a major role during development and it is commonly dysregulated in many diseases, including cancer. This highly concerted series of ligands, receptors, cytoplasmic signaling molecules, transcription factors, and co-regulators is involved in regulating the biological functions controlled by this pathway. Activation of Hh/GLI in cancer is most often through a non-canonical method of activation, independent of ligand binding. This review is intended to summarize our current understanding of the Hh/GLI signaling, non-canonical mechanisms of pathway activation, its implication in disease, and the current therapeutic strategies targeting this cascade.

**Abstract:**

The Hh/GLI signaling pathway was originally discovered in *Drosophila* as a major regulator of segment patterning in development. This pathway consists of a series of ligands (Shh, Ihh, and Dhh), transmembrane receptors (Ptch1 and Ptch2), transcription factors (GLI1–3), and signaling regulators (SMO, HHIP, SUFU, PKA, CK1, GSK3β, etc.) that work in concert to repress (Ptch1, Ptch2, SUFU, PKA, CK1, GSK3β) or activate (Shh, Ihh, Dhh, SMO, GLI1–3) the signaling cascade. Not long after the initial discovery, dysregulation of the Hh/GLI signaling pathway was implicated in human disease. Activation of this signaling pathway is observed in many types of cancer, including basal cell carcinoma, medulloblastoma, colorectal, prostate, pancreatic, and many more. Most often, the activation of the Hh/GLI pathway in cancer occurs through a ligand-independent mechanism. However, in benign disease, this activation is mostly ligand-dependent. The upstream signaling component of the receptor complex, SMO, is bypassed, and the GLI family of transcription factors can be activated regardless of ligand binding. Additional mechanisms of pathway activation exist whereby the entirety of the downstream signaling pathway is bypassed, and PTCH1 promotes cell cycle progression and prevents caspase-mediated apoptosis. Throughout this review, we summarize each component of the signaling cascade, non-canonical modes of pathway activation, and the implications in human disease, including cancer.

## 1. The History of Hedgehog

The first identified components of the Hh/GLI signaling pathway were fused and cubitus interruptus, the *Drosophila* homologue of the GLI family of transcription factors [1,2]. In 1980, Nusslein-Volhard and Wieschaus discovered the Hh ligand as part of their search for embryonic lethal mutants in *Drosophila.* The pair discovered 15 genetic loci implicated in segment patterning in *Drosophila.* When mutated, each locus produced distinct alterations in larvae patterning. The mutation in the Hh locus produced denticles on the body of the fly that were oriented in the opposite direction of non-mutants, indicating the importance of Hh in polarity and development in larvae. The resemblance of these mutants to a hedgehog inspired the name of this genetic locus. This study by Nusslein-Volhard and Wieschaus was foundational for the later characterization of the Hh signaling pathway, as they not only identified the Hh ligand but also identified Ptch1 as another Hh-related locus crucial for development [3].

Within the next two decades, scientists had thoroughly detailed the full DNA sequence of the Hh gene in *Drosophila* [4,5,6]. From this work, researchers found a remarkable sequence homology between the *Drosophila* Hh gene and that of vertebrates. First, the Sonic Hedgehog (Shh) ligand was characterized in chick embryos, and not long after, the three vertebral homologues, Shh, Indian Hedgehog (Ihh), and Desert Hedgehog (Dhh), were discovered [7,8]. It was not until the late 1990s that the GLI family of transcription factors was thought to be involved in the Hh signaling pathway [9,10,11,12,13]. Out of these discoveries came the thorough characterization of the Hh/GLI signaling cascade that we are most familiar with. GLI’s major negative regulator, suppressor of fused (SUFU), had already been discovered at this point, but its direct interaction with the GLI family of transcription factors was still unknown. In his study, Préat et al. investigated the phenotypic effects of the fused protein and potential suppressors of this phenotype. This investigational team discovered SUFU as the mitigator of the fused mutant phenotype. The fused mutant *Drosophila* have alterations in segment patterns and often have ovarian tumors, but overexpression of SUFU abrogated these phenotypes [14]. By the early 2000s, many components of the signaling cascade had been discovered, and their interactions with each other were becoming known.

Along with the increasing understanding of the Hh/GLI signaling pathway components came insights into its role in physiology. From the outset, alterations in segment patterning in *Drosophila* caused by aberrant Hh/GLI signaling indicated a significant role of the Hh/GLI pathway in development [1,2,3,14]. Nusslein-Volhard and Wieschaus observed that mutations in Hh caused loss of ventral and bilateral patterning in larvae [3]. Mutations in the Hh/GLI signaling pathway leading to disruptions in patterning which ultimately affected normal development were confirmed by Préat in his study of fused [14]. Continued studies into the role of Hh/GLI signaling in development confirmed that this pathway was conserved in vertebrates, highlighting the importance of this signaling pathway, particularly the necessity for highly regulated and timed activation of this signaling pathway to facilitate normal embryonic development. The first indications of the involvement of the Hh/GLI signaling pathway in the development of vertebrates were in studies of the morphogenesis of the craniofacial complex. Mutations in Shh caused midline patterning defects in developing embryos that led to holoprosencephaly and cyclopia. Further studies into the role of the Hh/GLI pathway in the development of the head uncovered this pathway as essential for development of the frontonasal and maxillary processes. Even transient loss of Shh in this context is sufficient to cause cleft lip/palate and mold holoprosencephaly [15,16,17,18]. In addition to craniofacial development, the broader role of the Hh/GLI signaling pathway in embryonic development was being discovered. Aberrations in this signaling pathway were now being linked to defects in the brain, spinal cord, skeleton, and limbs [18,19].

Evidence for a role of the Hh/GLI signaling pathway in human disease was not defined until the late 1990s. The first implication was in basal cell nevus syndrome in 1996 [20,21]. Characteristics of this syndrome include formation of basal cell carcinomas and developmental abnormalities usually involving the skin, further emphasizing the importance of this signaling cascade in development [20,21,22,23,24]. Though this syndrome is associated with the development of tumors, the direct implication of the Hh/GLI signaling pathway in cancer came later. At this point, the Hh/GLI signaling pathway was implicated mostly in non-cancer diseases, such as holoprosencephaly and other craniofacial developmental defects, abnormal skeletal development, aberrations in the nerve sheath, and infertility [25,26,27]. More recently, this signaling cascade has been characterized as a major contributor to a wide variety of cancers, such as basal cell carcinoma, medulloblastoma, colorectal, prostate, pancreatic, and others [28,29,30,31,32,33,34,35].

By the early 2000s, researchers were beginning to appreciate the complexity of the Hh/GLI signaling pathway, not only on a molecular level, but also in terms of disease relevance. The pathway consists of a highly concerted series of interactions between ligands, receptors, transducers, co-regulators, and transcription factors, with the potential to function both as an autocrine and paracrine signaling cascade. Further, the vast network of target genes suggested a degree of responsiveness to cellular context inherent to this signaling pathway. From the outset, there was a considerable degree of complexity to the Hh/GLI signaling pathway that left some wondering whether this cascade should be considered as part of a network instead of an isolated signaling pathway [36,37]. To date, there have been 40 genes identified as involved in the Hh/GLI signaling pathway in *Drosophila,* while there are 56 Hh/GLI signaling pathway genes in mammalians [38]. Pathway components are continuing to be discovered as we learn more about the mechanisms and functions of this pathway and its complex nature.

## 2. Hh/GLI Signaling Pathway Components

**Figure 1:**
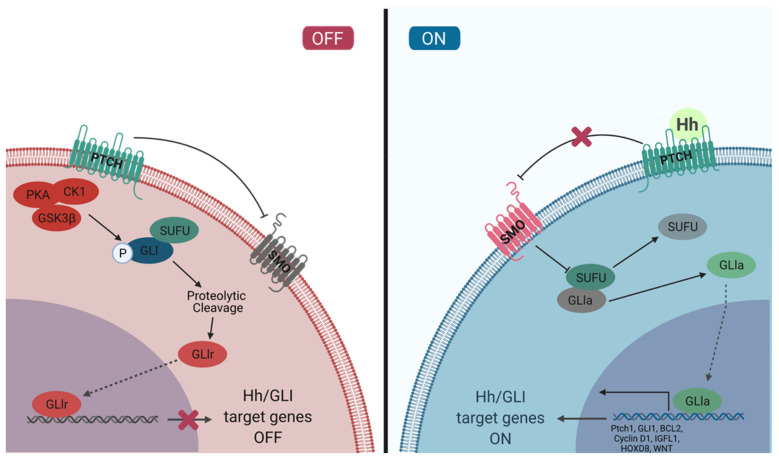
Activation of the Hh/GLI signaling pathway. Left panel: In the absence of ligand binding, PTCH exerts repressive effects on SMO. GLI transcription factors are sequestered by SUFU and phosphorylated by PKA, CK1, and GSK3β, marking them for proteolytic cleavage. The cleavage of the C-terminal domain creates GLIr, the repressor form of the transcription factor. GLIr then translocates into the nucleus and represses the transcription of Hh/GLI target genes. Right panel: Hh ligand binding to the extracellular domain of PTCH inhibits the receptor, relieving the repressive effects on SMO. SMO then inhibits the sequestration by SUFU and phosphorylation by PKA, CK1, and GSK3β, sparing GLI from proteolytic cleavage. The full-length form of GLI is a transcriptional activator that translocates into the nucleus and promotes the transcription of Hh/GLI target genes such as PTCH1, GLI1, BCL2, Cyclin D1, IGFL1, HOXD8, and WNT. The canonical Hh/GLI signaling pathway is most typically restricted to the primary cilium, however aberrant activation of this pathway may occur in alternate cellular compartments such as the cell membrane.

The Hh/GLI signaling pathway is a highly regulated, concerted cascade of extracellular ligands, receptor proteins, cytoplasmic signaling molecules, transcription factors, co-regulators, and target genes (Figure 1). The interactions between components of this signaling pathway are spatiotemporally regulated to ensure activation of the pathway only in proper cellular and tissue context. Typically, the Hh/GLI signaling pathway remains in the off state in mature, adult cells. The activation of this signaling cascade is required for tissue development and homeostasis, and has a significant role in the maintenance of pluripotent and somatic stem cell populations in the skin, mammary tissue, prostate epithelium, neural tissue, exocrine pancreas, and lung epithelium [39,40,41,42,43,44]. Hh/GLI signaling is also temporarily activated in wound healing and tissue repair, where stem cell populations are actively contributing to tissue production [45]. This pathway is tightly regulated and repressed in developed tissues, and dysregulation can lead to developmental disorders and disease.

### 2.1. Extracellular Ligands

In vertebrates, the Hh/GLI signaling pathway can be activated by Shh, Ihh, or Dhh. These extracellular ligands are lipid-modified proteins which, although they share high N-terminal sequence identity (76–91%), carry out different developmental functions [46]. Dhh had the lowest sequence identity to either Shh or Ihh. Its function is typically associated with development of gonad tissue, specifically ovarian granulosa cells and testicular sertoli cells [47,48,49]. Shh and Ihh, which exhibit the highest sequence homology, have some shared functions in several tissues, yet their role in developmental regulation is mostly unique. Ihh is a major regulator of skeletal development, particularly in endochondral ossification [8,50,51]. Shh is critical in patterning in development, specifically implicated in dorsal-ventral neural tube patterning, anterior-posterior limb patterning, and brain, teeth, and foregut development [52,53,54,55,56,57]. Though similar, each ligand promotes tissue-specific and highly regulated activation of the Hh/GLI signaling pathway.

Perhaps the most unique characteristic of the Hh ligands is the post-translational modifications, including the addition of a cholesterol molecule to the N-terminal domain which is required for activation and transport of the ligand between cells [58,59,60]. The Hh ligands are originally produced as precursor molecules [60,61,62]. First, approximately 25 amino acids are cleaved from the N-terminal domain of the 46 kDa precursor molecule, removing the signal peptide. Then, the remaining protein is further cleaved into the 19 kDa N-terminal fragment (N-Hh) and the 25 kDa C-terminal fragment (C-Hh) [25,62]. This cleavage is mediated by an internal, autoproteolytic mechanism [25,61,62]. The C-terminal fragment has catalytic activity, whereby a cystine residue initiates a neutrophilic attack on the neighboring glycine residue in the N-terminal fragment. This leaves a thioester, which is then attacked by a cholesterol, resulting in the covalent attachment of a cholesterol group to the C-terminal domain of N-Hh [25,58]. The cholesterol modification associates with the lipid in the cell membrane, tethering N-Hh to the outside of the cell [25,58,59]. This association with the cell membrane facilitates the final processing step of the N-Hh ligand: palmitoylation of the N-terminal domain by skinny Hedgehog acyltransferase (Ski) [58,63,64]. Not only is the cholesterol group important in facilitating the palmitoylation modification, but it has also been implicated in stabilizing N-Hh and facilitating long-range transport of N-Hh for paracrine signaling [65,66,67,68]. Now that N-Hh is dually modified, it is a fully activated signaling molecule. However, it is still tethered to the cell membrane of the secreting cell. The protein, Dispatched (Disp), is responsible for releasing the tethered ligand from the cell membrane as the final step in secreting the Hh ligand for activation of the signaling cascade [25,58,69,70].

Though the fully modified ligand has been secreted, the simple release of the tethered ligand is not enough to create the highly regulated morphogen gradient that is required for N-Hh to regulate development. For this, additional proteins and enzymes are required to chaperone the diffusion of the ligand. The heparan-sulfate-synthesizing enzyme, EXT, is required for the movement of N-Hh [25,58,71,72,73,74,75]. Heparan sulfate proteoglycan (HSPG) can by synthesized by EXT and localize to the cell surface, which can interact with N-Hh and lipoprotein lipophorin to facilitate the loading of Hh into soluble lipoproteins to facilitate the trafficking of the ligand [76,77]. An additional enzyme involved in mediating the release of N-Hh from the cell surface is Sulfatase1 (SULF1). Through its desulfation activity, SULF1 can disrupt the interaction between HSPGs at the cell surface. This can work to both repress or facilitate Hh/GLI signaling. SULF1 activity on the cell surface of the secreting cell will facilitate the release of N-Hh to stimulate signaling, whereas SULF1 activity on the cell surface of the receiving cell will disrupt ligand binding and repress the Hh/GLI signaling pathway [77,78]. N-Hh is characterized as the signaling domain, and through its interactions with various proteoglycans, enzymes, and lipoproteins, it will ultimately bind the cell surface receptors of the receiving cell to initiate Hh/GLI signaling.

### 2.2. Receptor Complex

The Hh/GLI signaling pathway receptors are localized to the primary cilia (PC) in most tissues. This microtubule-based organelle emerges from the cell surface and is implicated in mediating and interpreting mechanical, chemical, and thermal signaling [79,80,81]. The role of the PC as the Hh/GLI signaling transduction hub is conserved from invertebrates to vertebrates [61,62,63,64]. The transmembrane Hh ligand receptor, Patched (Ptch), is located at the base of the PC [82]. In vertebrates, there are two homologs of this receptor, Patched 1 (PTCH1) and 2 (Ptch2). In the absence of Hh ligand binding, these two receptors are responsible for repressing the activity of Smoothened (SMO), a transmembrane signaling protein [83]. PTCH1 extracellular domain contains a sterol-sensing domain (SSD) which interacts with the cholesterol modification of the Hh ligands [84]. When Hh ligands are bound to PTCH1, the receptor is internalized and trafficked to the lysosome for degradation, thereby relieving its repressive effects on SMO [85,86].

In the absence of PTCH1, SMO is phosphorylated by CK1α and G protein-coupled receptor kinase 2 (GRK2). This activates SMO and promotes its translocation into the PC [87,88]. SMO translocation is mediated by β-arrestin and Kinesin-like protein Kif3A, which interact with kinesin family member 7 motor protein (Kif7) for transport [89,90,91]. The SMO-β-arrestin complex inhibits both cAMP-dependent PKA and CK1, blocking the phosphorylation and proteolytic cleavage of GLI2/3. The full-length GLI proteins are active and will translocate into the nucleus to promote transcription of Hh/GLI target genes [92,93,94,95]. Additional studies have shown that SMO is the source of an additional signal through engaging GTP-binding regulatory proteins (G protein) [96,97,98,99]. SMO can activate the G_i_ family of G proteins, which, in some cellular contexts, is required for the activation of GLI transcription factors [96,98,99]. The involvement of G proteins in activating GLI transcription factors provides a partial explanation as to how Hh/GLI signaling can regulate such a wide variety of cellular functions. In recent years, SMO has also been shown to bind cholesterol, which activates the receptor and contributes to the Hh/GLI signal activation [100,101]. It is yet to be uncovered whether cholesterol binding is required for canonical activation of the Hh/GLI signaling pathway or if this is yet another mode through which Hh/GLI signaling can be activated, independent of the ligands.

PTCH1 is primarily expressed in mesenchymal cells, with Shh produced in neighboring epithelial cells, while Ptch2 is expressed mainly in testicular and skin epithelial cells [102,103]. Though both PTCH1 and Ptch2 are capable of binding all three Hh ligands, PTCH1 is considered to be the primary receptor [104]. Additionally, co-receptors have been identified that can modulate the activity of the Hh/GLI pathway. Boc, Cdon, and Growth-arrest-specific-1 (Gas1) can interact with the Hh ligands to ultimately activate the Hh/GLI signaling pathway [105,106,107]. On the other hand, co-receptors such as Hedgehog-interacting protein (Hhip) can negatively regulate the Hh/GLI signaling pathway. Hhip is a membrane glycoprotein that can bind all the Hh ligands, which prevents their interaction with PTCH1 and ultimately attenuates the signal [108]. Hhip is a target of the Hh/GLI signaling pathway and serves as part of the negative feedback loop, maintaining the balance of Hh/GLI signal activity [35,108]. Together, these receptors can increase or decrease Hh/GLI signaling, and are thought to regulate the level of activation of the Hh/GLI signaling cascade [109].

### 2.3. The GLI Family of Transcription Factors

Triggering of the Hh/GLI signaling pathway results in the activation of the GLI family of transcription factors to translate the extracellular ligand binding into a gene expression response. The GLI family of transcription factors can interact with each other and a variety of co-regulators to regulate specific subsets of gene targets based on those interactions. Though GLI1–3 have high sequence homology, they exhibit differences in tissue expression, transcriptional regulation, and binding partners. Not only does this increase the complexity of the signaling cascade, it also helps to explain how a single signaling cascade can have such a wide variety of transcriptional responses based on cellular and tissue context.

**Figure 2:**
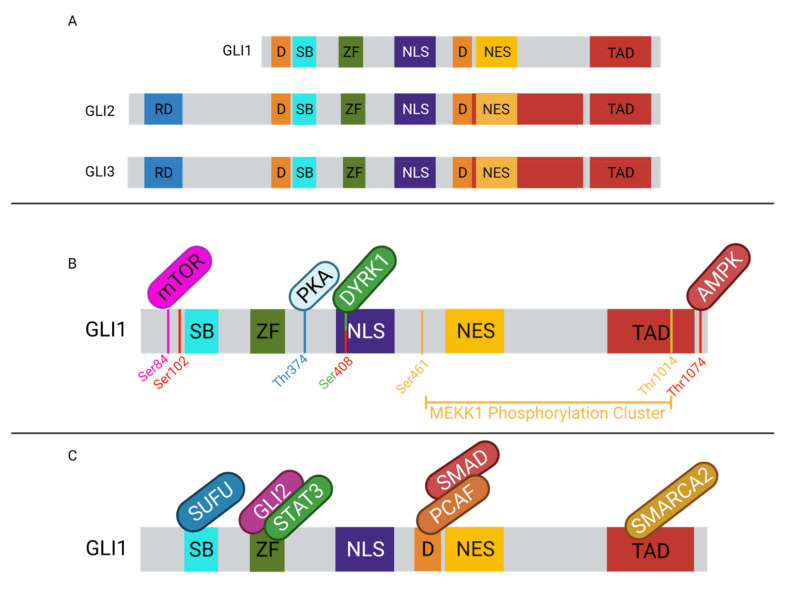
GLI1–3 regulatory and protein-interacting domains, post-translational modification sites, and interactions with co-regulators. (**A**) GLI1–3 proteins and their relevant regulatory sequences and protein-interacting domains. RD (blue) represents the repressor domain, D (orange) represents degron sequence, SB (cyan) represents the SUFU binding site, ZF (green) represents the zinc finger domain, NLS (purple) represents the nuclear localization signal, NES (yellow) represents the nuclear export signal, and TAD (red) represents the transcriptional activator domain. (**B**) GLI1 protein amino acids required for post-translation modifications. Serine 84 is recognized and phosphorylated by mTOR (pink). Serine 102, Serine 408, and Threonine 1074 are recognized and phosphorylated by AMPK (red). Threonine 374 is recognized and phosphorylated by PKA (blue). Serine 408 is also recognized and phosphorylated by DYRK1A (green). MEKK1 (yellow) recognizes and phosphorylates a series of amino acids between Serine 461 and Threonine 1014. (**C**) GLI1 protein-interacting domains for several co-regulators. SUFU (blue) binds the SUFU binding domain. GLI2 (purple) and STAT3 (green) can complex and bind to the zinc finger domain. PCAF (orange) and SMAD2/4 (red) can bind near the degron. SMARCA2 (yellow) can bind in the trans-activator domain.

The GLI family of transcription factors are a subfamily of the Krüppel family of transcription factors, which share a highly conserved zinc-finger domain for DNA binding [94,110,111]. GLI1–3 contain a 5-finger domain towards the center of each protein. Fingers 2–5 will recognize and bind the GACCACCCA motif, contacting the major groove and wrapping around the DNA. Finger one does not contact the DNA [112]. It is thought that the zinc fingers may also mediate protein–protein interactions [113]. Additionally, each of the GLI proteins has a nuclear localization signal (NLS), a nuclear export signal (NES), and SUFU binding domains [94]. GLI1 contains a single activation domain in the C-terminus and GLI2/3 contain 2 activation domains in the C-terminus, which interact with transcriptional activators to propagate the transcriptional activation effects of the GLI family of transcription factors (Figure 2) [94,114,115]. While GLI1–3 all contain C-terminal transactivation domains [94,116], only GLI2/3 contain an N-terminal repression domain, which contributes to their activity as transcriptional repressors [10,94,114]. The GLI proteins exhibit predominantly nuclear localization [111,117], and utilize both NLS-dependent and independent mechanisms. GLI2/3 each contain two NLS, one which is dependent on nuclear import machinery importin α/β, while the other NLS is independent of this machinery and instead interacts with importin β2 [94,118,119]. The SUFU binding domain, SYGH, is common amongst GLI1–3 and is required for negative regulation by SUFU [118,120,121,122]. SUFU binding to this motif prevents recognition of the NLS on GLI, supporting the cytoplasmic sequestration of GLI [118,120,121,122].

GLI protein activity is regulated through various mechanisms. Internal regulatory sequences, post-translational modifications, and co-regulatory proteins all have a role in modulating GLI activity (Figure 2). GLI2/3 contain destruction signals, one localized to each terminus, that promote the rapid degradation of the protein and prevent cytoplasmic accumulation. The rapid turnover of the proteins prevents GLI transcriptional activation [123]. A specific set of post-translational modifications (PTMs) mark GLI2/3 for proteasomal degradation. In the Hh-off state, GLI2/3 will be phosphorylated by PKA on the P1-6 domain, which allows CK1 and GSK3β to recognize and further phosphorylate the proteins [94,114,123,124,125,126]. The phosphorylated GLIs can then be bound by βTrCP, which facilitates ubiquitination by SCFβTrCP and promotes proteasomal degradation. The C-terminal end of GLI2/3 is cleaved, removing the activation domain and facilitating the transition into the repressor form [10,30,124,125,127]. When Hh/GLI signaling is active, GLI2/3 are not phosphorylated in this domain and are spared from proteasomal degradation. The proteolytic processing of GLI2 is not as efficient as it is for GLI3, therefore GLI2 is typically found in its full-length form [124]. GLI3 be found in the full-length form contributing to tumorigenesis, however not as often as GLI2 [128]. Similarly, GLI1 is not majorly regulated by proteasomal degradation. Instead, GLI1 activity is regulated at the transcriptional level and by cytoplasmic sequestration [10,116]. GLI1 is typically not expressed in Hh-off cells, but when the signal is perpetuated by GLI2/3, in their active forms, it can induce the expression of GLI1 to amplify the effects of Hh signaling [94,116,127]. Given that the proteolytic cleavage is most efficient in GLI3, it is not surprising that GLI3 is typically seen as the transcriptional repressor, while GLI1/2 are suggested to be the main transcriptional activators of the pathway [114,116].

In addition to proteasomal degradation, GLI proteins can also be regulated through protein–protein interactions (Figure 2). GLI1–3 can bind with a variety of proteins, cytosolic and nuclear, which can affect their transcriptional activity. The most common mechanism of GLI repression is through SUFU binding. This negative regulator is thought to sequester GLI1–3 in the cytoplasm to prevent nuclear translocation and further transcriptional activation of Hh/GLI signaling pathway target genes [129]. Similarly, MEKK1 can phosphorylate the C-terminal domain of GLI1, promoting its association with 14-3-3, a cytoplasmic protein, thereby sequestering it in the cytoplasm and inhibiting nuclear translocation [130]. Another GLI protein interaction is with Missing in Metastasis (MIM), which can interact with the SUFU-GLI complex and facilitate the release of GLI1–3, enhancing transcriptional activation [131,132]. Additional proteins such as aPKC can bind with GLI-bound MIM complex and can further phosphorylate GLI1, leading to maximal DNA binding affinity and therefore maximizing the transcriptional activation via GLI1 [131]. Similarly, mTOR can promote GLI1 dissociation from SUFU by phosphorylating it, which simultaneously promotes nuclear translocation and enhances GLI1 activity [133]. The GLI proteins can also interact with other nuclear proteins to enhance transcriptional activation. GLI2/3 can interact with CBP/p300 via their C-terminal domain to promote transcription [134,135,136]. The histone acetyltransferase, PCAF, can interact with GLI1 in the promoter of target genes to promote open chromatin conformations and help facilitate transcription [137]. This PCAF–GLI1 interaction has also been observed in TGFβ signaling with the addition of SMAD2/4 to the complex [138]. Interestingly, PCAF may also negatively regulate the activity of GLI1 through its ubiquitin ligase activity. PCAF interaction with non-DNA-bound GLI1 will result in ubiquitination of GLI1, ultimately targeting it for proteasomal degradation and inactivation [139,140]. SMARCA2 is yet another known co-activator of GLI1. The direct protein–protein interaction can facilitate an open chromatin conformation to promote transcriptional accessibility of target genes [141]. Similarly, SMARCA4 has been shown to interact with GLI1/3 to activate gene transcription and to interact with GLI3R to repress gene transcription through mediating an open or closed chromatin conformation, respectively [142]. Through their transactivation domain, GLI1/2 can interact with TAF9, a transcriptional co-activator, to enhance their transcriptional activity [143,144]. GLI1 has also been shown to interact with DYRK1A, which can enhance GLI1 transcriptional activity through two different mechanisms. First, DYRK1A can retain GLI1 in the nucleus [145], and DYRK1A may also phosphorylate GLI1, positively regulating its transcriptional activity [94,116]. DYRK2 carries out the opposite effect on GLI2, where phosphorylation of GLI2 targets the protein for proteasomal degradation, producing the repressor form of GLI2 [94]. Similarly, AMPK can phosphorylate GLI1 and target the protein for degradation, thereby inhibiting transcriptional activity. Within the nucleus, when GLI2/3 bind DNA in a repressive form, they can recruit histone deacetylases (HDAC) to the N-terminal domain to promote chromatin remodeling and gene silencing in that region [94]. Interestingly, GLI interactions with HDACs have also been shown to mediate GLI induction of target genes as well, acting as positive regulators of GLI function [142,146]. GLI1 and GLI3 can interact with SOX9 in the promoters of genes involved in chondrocyte differentiation, and this interaction can both activate or repress target gene transcription based on cellular context [147]. In addition to the aforementioned interactions with co-activators and repressors, the GLI family of transcription factors can form dimers to regulate the transcription of a subset of Hh-responsive genes. GLI1 and GLI2 have been shown to physically interact, and depletion of GLI1 can inhibit GLI2 occupancy in some promoters (BCL2, MYCN, and CCND1), indicating that this interaction may be required for activation of a subset of GLI-regulated genes [148]. The protein interactions and PTMs are numerous, each impacting GLI activity in a unique way and supporting the adaptability of this signaling pathway to cellular context.

The major negative regulator of the GLI family of transcription factors, SUFU, is a 484 amino acid protein that acts as a negative regulator of GLI1–3 [149]. SUFU can act as an adaptor protein, binding GLI1–3 and interacting with additional components of the cytoskeleton to sequester GLI or interacting with kinases to promote PTMs of GLI. It has also been suggested that SUFU acts as a chaperone in GLI1–3 nuclear translocation. Taken together, SUFU has a determining role in the cellular localization of GLI1–3, and this is the main mechanism through which SUFU carries out its regulatory effects on the GLI proteins [150]. Increasing evidence to support both theories suggests that SUFU may act through different mechanisms to regulate GLI activity [118,151,152,153]. SUFU localizes to both the cytoplasm and nucleus, and a recent study showed two of the mechanisms by which SUFU represses GLI1/2. For GLI1, SUFU piggybacks off of the CMR1-mediated nuclear export of GLI1 to promote cytoplasmic localization of GLI1 and repress its activity. For GLI2, SUFU uses a cytoplasmic tethering mechanism [154]. The latest characterization of the full-length SUFU demonstrated that this protein can alternate between an open and closed state, which is influenced by the presence or absence of GLI binding. The alternation between these states is conserved between human and *Drosophila* SUFU protein, indicating that this behavior is important for SUFU function. The N- and C-terminal domains of SUFU will come together to “sandwich” GLI and promote a stable interaction. It is suggested that GLI might mediate SUFU’s conformational change from the open to closed state, interacting with both the N- and C-terminal domains and dragging them together. The closed state predominates when SUFU is negatively repressing GLI activity, and this conformation is inhibited when Hh/GLI signaling is active. GLI dissociation is promoted by Hh signaling, and SUFU takes on the open conformation, yet the mechanistic details of this dissociation are still unknown [153].

SUFU is an adaptor protein and interacts with both cytoplasmic and nuclear proteins. Most of the SUFU protein–protein interactions are with other negative regulators of transcription, however, there have been a few reports of SUFU interacting with positive regulators of transcription, though the nature of those interactions has not been fully characterized. SUFU can interact with SAP18, a component of the Sin3 co-repressor complex that recruits HDACs to remodel chromatin in promoter regions and decrease gene expression. SUFU, SAP18, and Sin3 cannot bind the DNA, but require the SUFU–GLI interaction to be recruited to GLI-responsive promoters [154,155,156]. SUFU can also interact with an E3 ligase, Skp1-Cul1, and an F-box protein, Fbxl17, which ubiquitinate SUFU upon Hh signaling and facilitate SUFU degradation within the nucleus [157]. This relieves SUFU repression of GLI and promotes transcriptional activation. SUFU interaction with PKA and GSK3β can prevent its nuclear degradation and stabilize Hh/GLI signaling. It was recently discovered that SUFU can interact with protein phosphatase 4 regulatory subunit 2 (Ppp4r2) to dephosphorylate it, leaving it susceptible to degradation via Skp1-Cul1 and Fbxl17, driving the turnover of SUFU in the nucleus [150]. Lastly, Galectin 3, a partner of pCIP, was identified as another SUFU-interacting protein, suggesting that SUFU might be involved in mRNA maturation [154]. pCIP also has acetyl transferase activity and can interact with CBP and p300, indicating that SUFU may also be involved in the recruitment of proteins which facilitate gene transcription [138,154]. Nuclear SUFU can also interact with various regulatory proteins, including p66β and Mycbp, which can negatively and positively regulate the functions of SUFU, respectively [158]. SUFU can interact with a variety of proteins which can influence its role as a repressor in the Hh/GLI signaling pathway.

The various degrees of cooperation between GLI1, 2, and 3 create a network of interaction commonly referred to as the GLI code. The interactions between these proteins are largely dictated by cellular context, and the repressor and activators can work together to create a spectrum of transcriptional responses [32,127]. Isoforms of each GLI protein have been characterized, and like GLI1–3, some of these isoforms exhibit additional tissue specificity. This contributes further to the various roles and cellular responses of Hh/GLI signaling, and promotes unique downstream effects based on tissue type or cellular context [128,159,160]. The GLI network is not yet fully understood, but there are a few contexts in which the interactions have been characterized relating to frog neuronal networks. Early studies of different combinations of GLI activity required for appropriate embryo patterning revealed that only GLI1 can induce floor plate differentiation, while only GLI2/3 were involved in skeletal patterning [114,161]. Further, GLI2/3 can inhibit GLI1 induction in the ventral forebrain and floor plate in developing frog embryos [162], while GLI3 can inhibit motoneuron induction by GLI2 in a different context [161]. These examples illustrate the context-dependence cooperation of GLIs that can ultimately affect GLI activity. The notion of a GLI network can help to explain some of the contradictory findings of GLI activity that we can otherwise not explain. This includes GLI3 having an alternate subset of gene targets in the presence or absence of GLI1, or GLI2/3 cooperation having opposite effects on GLI1 activity compared to GLI3 alone [32,127]. Further, GLI1 and 2 have some common targets, yet also have distinct subsets of gene targets, further supporting the idea of GLI cooperation ultimately affecting the transcriptional activity on target genes [163].

Another subfamily of the Krüppel family of proteins is the GLI-similar (GLIS) transcription factors. GLIS1–3 contain zinc-finger domains and are closely related to GLI1–3 [111,164,165]. Apart from the highly conserved zinc-finger domain, GLIS1–3 have relatively low sequence homology [164,166,167,168,169]. These proteins are localized to the primary cilium and can undergo nuclear translocation after activation via PTMs, similar to GLI1–3. Once inside the nucleus, GLIS1–3 can bind DNA through the zinc-finger domain and regulate transcription. GLIS can recognize and bind the GLI binding motif, which suggests the potential for interaction between these two subfamilies of Krüppel proteins [111,170]. When co-expressed, GLIS2 can inhibit reporter activity of GLI1 [170]. GLIS can act both as transcriptional activators and repressors, and have been shown to interact with CtBP1 to recruit histone modifiers and promote transcriptional repression [164]. GLIS are involved in a wide variety of cellular processes, including cell proliferation, apoptosis, differentiation, and development. The potential for crosstalk between GLIS1–3 and GLI1–3 increases the network of interaction described in the GLI code

### 2.4. Hh/GLI Signaling Pathway Target Genes

The Hh/GLI signaling pathway is implicated in development, and typically, the target genes of this pathway are cell-type- and context-dependent. Many of the general gene targets of this pathway are developmental regulators, such as FGF4, Pax6-9, ABCG2, and Hhip, cell cycle regulators such as CCND2 and CCNE1, apoptosis regulator, BCL2, transcription factors FOXM1 and N-myc, and Wnt pathway proteins, JAG1, SFRP1, and Wnt [4,108,171,172,173,174,175,176]. In stem cells, Hh/GLI signaling can induce genes such as BMI1, LGR5, CD44, and CD133 through crosstalk with other signaling pathways such as Wnt. To promote epithelial-to-mesenchymal transition (EMT), the Hh/GLI signaling pathway can upregulate genes such as SNAI1, SNAI2, ZEB1, ZEB2, TWIST2, and FOXC2 [177]. Additionally, the Hh/GLI signaling pathway can induce the transcription of PTCH1 and Ptch2 as a form of pathway regulation [178]. Through direct induction of gene expression and interaction with various pathways, the Hh/GLI signaling pathway has a wide range of gene targets that can regulate cell cycle, promote differentiation and proliferation, EMT, and many more functions.

## 3. Non-Canonical Activation of the Hh/GLI Signaling Pathway

Typically, canonical pathway activation refers to extracellular ligand (Shh/Dhh/Ihh) binding PTCH1 and relieving repression of SMO to activate the downstream signaling cascade [179,180]. Though vitally important in development, canonical pathway activation is not widely implicated in disease. Instead, a non-canonical activation of the Hh/GLI signaling pathway, whereby SMO is bypassed, is a typical driver in disease. Non-canonical Hh/GLI pathway activation typically refers to SMO-independent activation, but sometimes more broadly includes ligand-independent pathway activation [180]. While Ptch1 is considered the primary Hh ligand binding target, other targets include Boc, Cdon, and Gas1. These interactions can activate the Hh/GLI signaling pathway independent of the Ptch1 receptor, as mentioned in the previous section. In addition, these receptors also play a critical role in other signaling pathways, such as myogenesis, axon guidance, CREB, ERK, GPCR, and apoptotic pathways, just to name a few [105,106,107]. While these binding targets can be considered part of the non-canonical activation of the Hh/GLI signaling pathway, there are two main mechanisms by which the signaling cascade can be activated to induce non-canonical Hh/GLI signaling. Type I is a SMO-independent mechanism and type II is a SMO-dependent mechanism that bypasses the GLI family of transcription factors.

In type I non-canonical Hh/GLI pathway activation, PTCH1 does not interact with the SMO receptor. Instead, PTCH1 is involved in the regulation of cell cycle and apoptosis through the caspase protein, and Shh ligand binding to the extracellular domain of PTCH1 can influence these interactions. In the unbound state, PTCH1 interacts with phosphorylated cyclin B1 [181,182]. PTCH1 can also recruit caspase-3, which will cleave a portion of the C-terminal domain of PTCH1. This cleavage will release caspase recruitment domain family member 8 (CARD) and adaptor protein found and a half LIM domains 2 (FHL2)/DRAL. This leads to the activation of caspase-9, which ultimately triggers apoptosis. When Shh binds to the extracellular domain of PTCH1, it inactivates the receptor and prevents this caspase-mediated apoptosis [182]. Moreover, cyclin B1 is released from the intracellular domain of the PTCH1 receptor and facilitates cell proliferation [181,182]. Type II non-canonical pathway activation is SMO-dependent, but does not rely on the GLI family of transcription factors to propagate its effects. In addition to promoting the active form of the GLI family of transcription factors, SMO also has functional G-protein coupled receptor (GPCR) properties with selectivity to heterotrimeric G_i_ proteins, through which it carries out this type II non-canonical pathway activation [96,182]. When the Hh ligand binds and inactivates PTCH1, SMO is activated. SMO uses these G_i_ proteins to activate phosphoinositide 3-kinase (PI3K) kinase, small GTPases Ras homologous family member A (RhoA), and Ras-related C3 botulinum toxin substrate 1 (Rac1) [183]. The SMO-Rho cellular response is rapid and leads to the reconstruction of actin cytoskeleton along with stress fiber formation, tubulogenesis, and tumor-dependent angiogenesis [184]. In addition to activating PI3K, RhoA, and Rac1, G_i_ proteins also promote Ca^2+^ influx into the cytosol. The Shh-SMO-G_i_ protein cascade activates phospholipase C (PLC) production, which increases PI3K and promotes opening of calcium channels in the smooth endoplasmic reticulum membrane, leading to a cytosolic spike in Ca^2+^ [185]. This Ca^2+^ influx affects differentiation, proliferation, apoptosis, and the migration of both neural and neuronal precursor cells [186,187]. Additionally, partial SMO agonists have demonstrated the ability to uncouple the SMO-Ampk-Ca^2+^ signaling axis from the Hh/GLI signaling pathway and drive metabolic changes favoring the Warburg effect [188]. Together, the effects of type II non-canonical Hh/GLI signaling pathway activation can lead to cytoskeletal reconstruction, angiogenesis, and proliferative effects, contributing to the progression of cancer.

**Figure 3:**
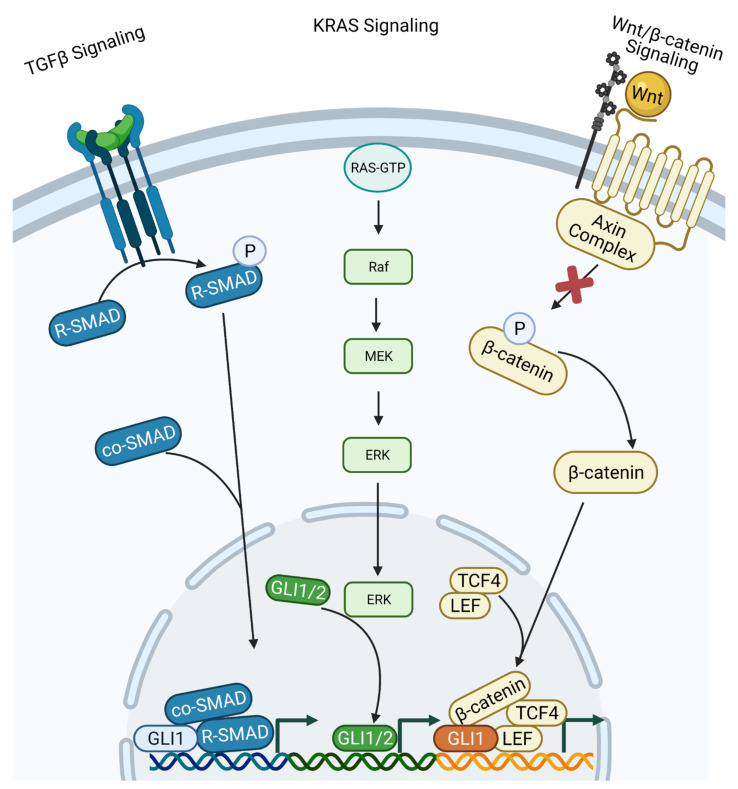
Non-canonical activation of the Hh/GLI pathway. TGFβ Signaling: Ligand binding in the extracellular domain induces the catalytic activity of the receptor on the intracellular domain. R-SMADs can be phosphorylated and complex with co-SMADs, then translocate into the nucleus. Inside the nucleus, SMADs can recruit and bind with GLI1 to activate the transcription of Hh/GLI target genes. KRAS signaling: Constitutively active KRAS will phosphorylate Raf, which in turn phosphorylates MEK, which then phosphorylates ERK. Activated ERK will then translocate into the nucleus and activate a variety of transcription factors, including GLI1/2. Wnt/β-catenin: Extracellular Wnt ligand binding promotes sequestration of the Axin complex to the intracellular side of the receptor. This inhibits phosphorylation of β-catenin, allowing for its nuclear translocation and recruitment of additional transcription factors, including GLI1, for transcriptional activation. Figure created with BioRender.

Crosstalk between other signaling pathways is another way in which the Hh/GLI pathway can be activated independent of ligand binding. There are many reports of the TGFβ, KRAS, and Wnt/β-catenin pathways activating the downstream constituents of the Hh/GLI pathway (Figure 3) [189,190,191,192,193,194,195,196,197,198,199,200,201]. The TGFβ signaling pathway is one of the most well-characterized pathways to interact with the Hh/GLI signaling. This cascade consists of a family of ligands which bind to TGFβ receptor II and facilitate its dimerization and recruitment of the TGFβ receptor I dimer to form an active tetramer. This complex phosphorylates their intracellular domains to activate kinase activity, recruits the regulatory SMADs, and phosphorylates and activates them. The regulatory SMADs then dissociate from the receptor complex and bind co-SMADs, which then translocate into the nucleus to promote transcription of TGFβ target genes (Figure 3) [198]. The TGFβ signaling pathway has dual roles in cancer depending on cellular context, acting as either a tumor suppressor or tumor supporter. In its role as a tumor suppressor, it can activate genes involved in cell cycle arrest and apoptosis through a SMAD4-dependent mechanism [199]. As a tumor supporter, this pathway can activate genes involved in immune suppression, angiogenesis, and EMT. Vimentin and cadherins can be induced to promote EMT, and genes such as VEGF and MMP9 are consistently upregulated by the TGFβ signaling pathway to promote angiogenesis [189,193,197,202,203]. Activation of the TGFβ signaling pathway will also lead to recruitment of inflammatory cells [189,193,197]. Through release of paused RNAPII [204,205,206,207,208,209,210,211,212], the TGFβ signaling pathway can induce the transcription of the GLI family of transcription factors and thereby increase their activity, leading to non-canonical activation of the Hh/GLI signaling pathway [196,204,213]. GLI1–3 have been well-characterized as direct transcriptional targets of the canonical, SMAD-dependent TGFβ signaling pathway [138,179,190,191,214]. This regulation of the GLI transcription factors is independent of SMO [190,215]. In addition to regulation at the transcriptional level, it has been shown that the TGFβ signaling pathway can interfere with PKA phosphorylation of GLI1–3, modulating their activity by preventing their proteasomal degradation [216]. Additionally, the GLI family of transcription factors can interact with the SMAD transcription factors, both increasing their activity and promoting transcription of an additional cohort of tumorigenic genes [138]. The crosstalk between the TGFβ signaling pathway and the Hh/GLI signaling pathway may be essential for the TGFβ signaling pathway to carry out its full, tumor-supportive effects [217,218,219]. Further, the interaction between these two pathways seems to be cyclical in that the activation of the Hh/GLI pathway by TGFβ can ultimately promote the expression of genes that activate the TGFβ signaling pathway [220,221].

The KRAS signaling pathway is another pathway that exhibits crosstalk with and activation of the Hh/GLI signaling cascade [180,200]. KRAS is a GTPase signal transducer protein that is commonly mutated in pancreatic ductal adenocarcinomas (PDACs), resulting in a constitutively active form of the protein. As such, KRAS is continually inducing the downstream signaling cascade Raf/MEK/ERK and promoting transcription of target genes (Figure 3). Active KRAS will recruit, phosphorylate, and activate RAF, which in turn will phosphorylate MEK. Active MEK can then bind and phosphorylate ERK to activate it and promote nuclear translocation. Inside the nucleus, ERK will activate transcription factors such and Jun or Fos to induce transcription of target genes [222,223,224]. Studies have shown that the constitutive activation of the KRAS signaling pathway can lead to increased GLI1 transcriptional activity [192,194,196]. Further investigations showed that MEK can activate GLI1 activity, promoting Hh/GLI signaling [225]. Additionally, GLI1 and GLI2 are transcriptional targets of the KRAS signaling pathway, similar to the TGFβ signaling pathway. Increased mRNA transcription will lead to increased protein translation and further accumulation of the proteins in the cytoplasm, eventually increasing their transcriptional activity [196,201].

The Wnt/β-catenin signaling pathway is a non-canonical activator of the Hh/GLI pathway through transcriptional regulation and protein–protein interactions. When Wnt signaling is off, cytoplasmic β-catenin is degraded through a similar mechanism as the GLI family of transcription factors. The Axin complex, which contains CK1 and GSK3β, will phosphorylate β-catenin in the absence of Wnt signaling, promoting the proteasomal degradation of β-catenin. The turnover of β-catenin in the cytoplasm prevents nuclear translocation, inhibiting the transcription of Wnt target genes. When Wnt binds to the Frizzled receptor, the receptor complexes with LRP6, and together, the complex recruits and sequesters the Axin complex, inhibiting its kinase activity. β-catenin can then accumulate in the cytoplasm, translocate into the nucleus, and bind either TCF or LEF to activate target genes (Figure 3) [226,227,228]. GLI2 transcription can be induced by β-catenin in a similar manner as the TGFβ signaling pathway [190,229]. Not only are GLI1/2 transcriptionally activated by β-catenin, but they can also form complexes with β-catenin in a similar manner as with the SMAD transcription factors [230]. Hh/GLI and Wnt/β-catenin pathways also share similar regulatory molecules, including SUFU, CK1, and GSK3β, indicating another mode for interaction [195,230]. There is some evidence to show that these two pathways may also interact in a cyclical manner, given that Wnt transcription can be activated by the GLI transcription factors [177,178]. Further, GLI3 can act in an inhibitor manner of some β-catenin targets, emphasizing that the interaction between these two pathways can be complex and bidirectional [231].

## 4. The Hh/GLI Signaling Pathway in Disease

The Hh/GLI signaling pathway is essential for regulating many developmental processes. It comes as no surprise that mutations, loss of function, or aberrant activation of the pathway can lead to developmental defects and disease [33,34,180,194]. For example, according to the Human Gene Mutation Database (HGMD), there are over two-hundred mutations (missense/nonsense, splicing, insertions/deletions, rearrangements, etc.) that can occur in the SHH gene that lead to deleterious effects. One of the most common diseases associated with mutations in the SHH gene is a developmental malformation known as holoprosencephaly [232]. In this disease, the brain is unable to divide properly into the right and left hemisphere. Severity of the disease can vary widely among effected individuals, with symptoms ranging from hypotelorism, microcephaly, hydrocephalus (causing macrocephaly) cleft palate, cyclopia, and proboscis [233]. Unsurprisingly, the physical distortion due to holoprosencephaly leads to other impairments, such as developmental delay, intellectual disability, seizures, inability to regulate temperature, and feeding difficulties, just to name a few [233]. Other developmental malformations due to Hh ligand mutations include radial hemimelia, polydactyly, cleft lip, bone development disease, and osteochondrodysplasia. Mutations in other pathway components such as PTCH1, SMO, and SUFU can lead to developmental malformations, including brachydactyly, Curry-Jones Syndrome, and Ellis-Can Creveld Syndrome, respectively [234].

While mutations play a crucial role in the aberrant Hh pathway leading to a plethora of developmental malformations, they can also lead to cancer. The pathway activators (Hh ligands, SMO, and GLI1–3) are considered proto-oncogenes due to their ability to upregulate the pathway and promote tumorigenesis. There are different mechanisms whereby the canonical Hh/GLI pathway can by dysregulated, leading to unwarranted activation. Ligand-independent activation of the signaling cascade increases the activity of GLI1–3, upregulating the expression of Hh/GLI pathway target genes. This type of pathway activation is commonly seen in Gorlin Syndrome, which is associated with the development of medulloblastomas, basal cell carcinomas, and rhabdomyosarcomas [20,235]. Additional mechanisms of aberrant pathway activation can be ligand-dependent, autocrine activation of the pathway, whereby cancer cells can upregulate the synthesis of the Shh ligand and stimulate Hh/GLI pathway activation. This type of pathway activation is common in prostate cancer, hepatocellular carcinoma, pancreatic cancer, and non-small cell lung cancer [236,237,238,239,240]. The Shh ligand can also be secreted by tumor cells and can participate in paracrine signaling, activating the Hh/GLI signaling pathway in both tumor and stromal cells, often seen in pancreatic and prostate cancers [34,235,241,242]. Lastly, tumor stroma can secrete the Shh ligand in a reverse paracrine mechanism, whereby the ligand will activate the Hh/GLI signaling pathway within tumor cells [243]. In some contexts, this paracrine signaling from the stroma can have a role in restraining tumor progression, further complicating the role of Hh/GLI signaling in cancer progression [241,242]. Each mechanism outlined above involves changes in the activity of Hh/GLI pathway constituents, classifying these as canonical mechanisms of pathway activation.

The repressors of the pathway (SUFU and PTCH1) are considered tumor suppressors, as their loss of function upregulates the Hh/GLI signaling activity to drive tumorigenesis [31,35,116,244,245]. It was not until recently that SUFU mutations were noted as potential drivers of disease. However, in a short time span, it has become increasingly clear that SUFU mutations have a significant impact in causing disease [23,24,30,244,246,247,248,249]. The involvement of the Hh signaling pathway in Gorlin Syndrome has been established previously, however, it was not until a study in 2018 that the contributions of SUFU mutations to this syndrome were brought to light. Gorlin Syndrome predisposes individuals to the development of basal cell carcinomas. Mutations in PTCH1 are linked to 85% of cases of Gorlin Syndrome, however a subset of 5% of cases exhibit deleterious mutations in SUFU. These mutations arise from an abnormal splice site which leads to loss of function of SUFU. Individuals with this mutation exhibit many characteristics of Gorlin Syndrome, but they also experience palmar sclerotic fibromas, which seems to be unique to this subset of patients. Further investigation into this subset of patients has revealed that SUFU gene mutations may actually cause a distinct cutaneous cancer predisposition syndrome that, while extremely similar to, is different from Gorlin Syndrome. Additional studies would be required in order to validate this observation [244]. Meningiomas resultant of Gorlin Syndrome or Familial multiple meningioma and childhood medulloblastomas have also been associated with SUFU mutations [244,246]. Additional studies in meningiomas identified a germline point mutation in SUFU present in a subset of patients. This mutation impacted the tertiary structure of SUFU, thereby impacting its tumor-suppressor function [246]. A nonsense mutation in SUFU was discovered in a subset of patients with nevoid basal cell carcinomas (as in Gorlin Syndrome). This mutation decreased the tumor-suppressor function of SUFU, similar to that in the subset of meningiomas previously described [23]. A similar cancer subtype, Merkel cell carcinoma, has been associated with SUFU mutation as a contributor to disease progression [30].

A study of patients with multiple hereditary infundibulocystic basal cell carcinoma syndrome was conducted to determine the role of SUFU mutations in disease progression. Individuals with the germline splice mutation resulted in not only the abolishment of SUFU protein, but it was also enough to place the individuals at higher risk for hereditary infundibulocystic basal cell carcinoma syndrome [24]. Another study determined the effects of both germline and somatic mutations in SUFU, and found that children with a splice mutation (causing a deletion of exon 6) presented with facial papules and dysmorphology due to this non-functioning SUFU protein [247]. In pancreatic cancer, a germline mutation in SUFU was found associated with intraductal papillary mucosal neoplasms (IPMNs) and elevated risk of pancreatic carcinomas. Given the correlation of increased IPMNs subsequently leading to PDAC, researchers found that patients with IPMNs in addition to a subset of germline mutations, including this SUFU mutation, were at higher risk of further developing pancreatic carcinomas than those with IPMNs and none of the identified germline mutations [248]. Lastly, a transcript variant of SUFU that contained a new and additional protein coding exon was identified in patients with PDAC. This mutation was correlated with increased metastasis in these PDAC patients [249].

In addition to the canonical activation of the Hh/GLI signaling pathway, non-canonical mechanisms of pathway activation have been implicated in disease. As previously described, the KRAS signaling pathway can result in overexpression of Hh/GLI target genes through interaction with and activation of GLI transcription factors. This non-canonical Hh/GLI pathway activation has been observed in many types of disease, including pancreatic ductal adenocarcinoma, melanoma, gastric cancer, colon cancer, lung cancers, multiple myelomas, and many more [192,196,201,250,251,252,253,254]. The TGFβ signaling pathway can similarly induce the expression of Hh/GLI target genes through SMAD transcription factor association with and activation of GLI transcription factors. Additionally, GLI1/2 transcription are induced by the TGFβ signaling pathway, ultimately leading to increased expression of Hh/GLI target genes. This TGFβ-mediated pathway activation is noted in pancreatic cancer, gastric cancer, and colon cancer, to name a few [138,190,255,256]. Lastly, we discussed the role of Wnt/β-catenin in promoting the transcription of Hh/GLI pathway target genes. This type of pathway activation is common in basal cell carcinomas, intestinal cancer, colon cancer, leukemia, brain tumors, ovarian cancer, and many more cancer subtypes [178,198,219,228,230,257,258].

This profound effect in tumor biology has triggered the development of Hh/GLI inhibitors for cancer treatment. Presently, most therapies in targeting the Hh pathway have been focused on developing SMO inhibitors (SMOi). Some of the therapeutics include sonidegib, vismodegib, saridegib, BMS-833923, taladegib, and glasdegib [259,260]. There has been some success in treating basal cell carcinoma, myeloid malignancies, medulloblastoma, and a few other advanced solid tumors using SMOi therapeutics [261,262,263,264,265,266]. Unfortunately, the rapid accumulation of mutations in cancer has created a resistance to SMOi treatments, typically through acquisition of the D437H mutation in SMO [267,268]. This acquired SMOi resistance has given rise to research investigating other oncogenic drivers as potential therapeutic targets. This redirection of research led to downstream Hh activators such as GLI as potential therapeutic targets. In 2007, Lauth et al. showed via in vitro and in vivo models that molecules GANT61 and GANT58 were able to block DNA binding of GLI and therefore reduce proliferation and tumor growth [269]. Another molecule, Glabrescione B, was also tested and found to interfere with GLI1 ability to bind to DNA, and therefore inhibited the growth of Hedgehog-dependent tumors [270]. Researchers also explored Hedgehog pathway inhibitors (HPIs), such as HPI1, HPI2, HPI3, HPI4, and arsenic trioxide (ATO) [271,272,273]. Interestingly, each of the HPI’s were observed to target different mechanisms of the pathway, with HPI1 inhibiting endogenous and exogenous GLI1/GLI2 activity [271], while HPI2 and HPI3 were observed blocking the ability of full-length GLI2 conversion into transcription activators [271], leaving HPI4 to target the cilia, ultimately disrupting ciliogenesis from occurring, which inhibits proper ciliary progression and trafficking that is required for Hh/GLI function [271]. Lastly, ATO was observed to inhibit GLI1 protein function in the nucleus, while also reducing GLI2 ability to traffic and accumulate to the cilia, resulting in reduced Hh activity [272,273]. Despite GLI inhibitors demonstrating reduced proliferation or tumor growth through their interference of DNA binding, trafficking, processing, accumulation, or activation of GLI, they were unfortunately short-lived due to significant cytotoxicity observed in in vitro studies. Research is now turning to alternative pathways that focus on the non-canonical GLI activation molecules. Combinations of SMOi and additional pathway constituent inhibitors provide the most hope of impairing Hh/GLI pathway activation in solid tumors and preventing acquired resistance [260].

## 5. Concluding Remarks

The Hh/GLI signaling pathway is a highly regulated complex cascade of ligands, receptors, transcriptional effectors, and regulatory proteins. Its initial discovery in *Drosophila* provided the first insights into its role in regulating development. Aberrations in the Hh/GLI signaling pathway can lead to developmental defects and disease, including cancer. Further studies into the pathway have demonstrated that not only mutations lead to increased Hh/GLI signaling observed in disease, but there is also an additional subset of pathway interactions and non-canonical mechanisms of pathway activation that are significant contributors to the altered activity observed in disease.

## Data Availability

Data sharing not applicable. No new data were created or analyzed in this work.
